# Open abdominal management after ruptured abdominal aortic aneurysm repair: from a single-center study in Japan

**DOI:** 10.1007/s00595-022-02574-9

**Published:** 2022-08-19

**Authors:** Yasumi Maze, Toshiya Tokui, Teruhisa Kawaguchi, Masahiko Murakami, Ryosai Inoue, Koji Hirano, Keita Sato, Yoshihisa Tamura

**Affiliations:** 1 Department of Thoracic and Cardiovascular Surgery, Ise Red Cross Hospital, 1-471-2 Funae, Ise, Mie 516-8512 Japan; 2 Department of Surgery, Ise Red Cross Hospital, Ise, Mie Japan

**Keywords:** Ruptured abdominal aortic aneurysm, Abdominal compartment syndrome, Open abdominal management

## Abstract

**Purpose:**

We investigated the utility of the open abdominal management (OA) technique for ruptured abdominal aortic aneurysm (rAAA).

**Methods:**

Between January 2016 and August 2021, 33 patients underwent open surgery for rAAA at our institution. The patients were divided into OA (*n* = 12) and non-OA (*n* = 21) groups. We compared preoperative characteristics, operative data, and postoperative outcomes between the two groups. The intensive care unit management and abdominal wall closure statuses of the OA group were evaluated.

**Results:**

The OA group included significantly more cases of a preoperative shock than the non-OA group. The operation time was also significantly longer in the OA group than in the non-OA group. The need for intraoperative fluids, amount of bleeding, and need for blood transfusion were significantly higher in the OA group than in the non-OA group. Negative pressure therapy (NPT) systems are useful in OA. In five of the six survivors in the OA group, abdominal closure was able to be achieved using components separation (CS) technique.

**Conclusions:**

NPT and the CS technique may increase the abdominal wall closure rate in rAAA surgery using OA and are expected to improve outcomes.

## Introduction

As a treatment strategy for severe trauma, Rotondo et al. advocated the concept of damage control surgery [1]. Subsequently, based on the idea of damage control surgery, open abdominal management (OA) has been used for the management of severe trauma [2–5] Treatment with OA has been used to prevent abdominal compartment syndrome (ACS) in the field of aortic surgery, and it contributes to the improvement of surgical outcomes [6–9]. When OA is performed, the definitive abdominal closure timing and method of abdominal closure are associated with the patient’s prognosis [4, 10, 11]. Many studies have reported that negative pressure therapy (NPT) is effective for temporary abdominal closure management [11–14]; however, the ideal definitive abdominal closure method is controversial. In particular, if the duration from the first surgery to definitive abdominal closure is long, the abdominal closure rate may decrease [10–12].

The components separation (CS) technique has been used for the repair of complex midline ventral hernia [15, 16]. A previous study reported that the CS technique can be used for definitive abdominal closure in OA [17]. At our institution, we have used the CS technique in addition to NPT to enable definitive abdominal wall closure, even in long-term cases of OA.

We, therefore, investigated the use and success rate of OA for ruptured abdominal aortic aneurysm (rAAA) at our institution.

## Methods

### Study design and patients

All operations and data collection were performed at the Ise Red Cross Hospital, Ise, Japan. Clinical outcome data were obtained from the hospital’s patient records or from the patient’s family doctor. This study was approved by the Institutional Review Board of Ise Red Cross Hospital (approval number ER2021-34), and the need for informed consent was waived owing to the retrospective nature of the study. All methods were performed in accordance with relevant guidelines and regulations.

In 2016, we introduced OA for rAAA surgery cases. Between January 2016 and August 2021, 48 patients underwent surgery for rAAA at our institution. Of the 48 patients, 12 underwent endovascular aortic repair (EVAR), 3 underwent extra-anatomical bypass because of an infectious aneurysm, and 33 who underwent open surgery were included in this study. The patients were divided into OA (*n* = 12) and non-OA (*n* = 21) groups. The preoperative characteristics, operative data, and postoperative outcomes of the two groups were compared. Furthermore, the intensive care unit (ICU) management and abdominal wall closure status of the OA group were evaluated.

### Operative technique

The choice of surgical procedure for each case was determined by a vascular surgeon who was familiar with EVAR. Since EVAR was performed by a radiologist at our hospital, even if EVAR was indicated, open repair was performed if the radiologist could not visit the hospital immediately. Therefore, the surgical procedure was selected according to the patient’s general condition, operating room preparedness, and arrival time of the vascular surgeon and radiologist [18].

#### Definition

Open repair was defined as the in situ replacement of an abdominal aneurysm with an artificial graft. OA was defined as the method of closing the abdomen in two phases. In this study, the abdomen remained open to prevent ACS. Preoperative shock was defined as a systolic blood pressure of < 80 mmHg. Door-to-procedure time was defined as the time from arrival at the hospital to the time of skin incision. The duration of OA was defined as the time from the initiation of OA until definitive abdominal closure was achieved.

### Indication and procedure for OA

**Fig. 1:**
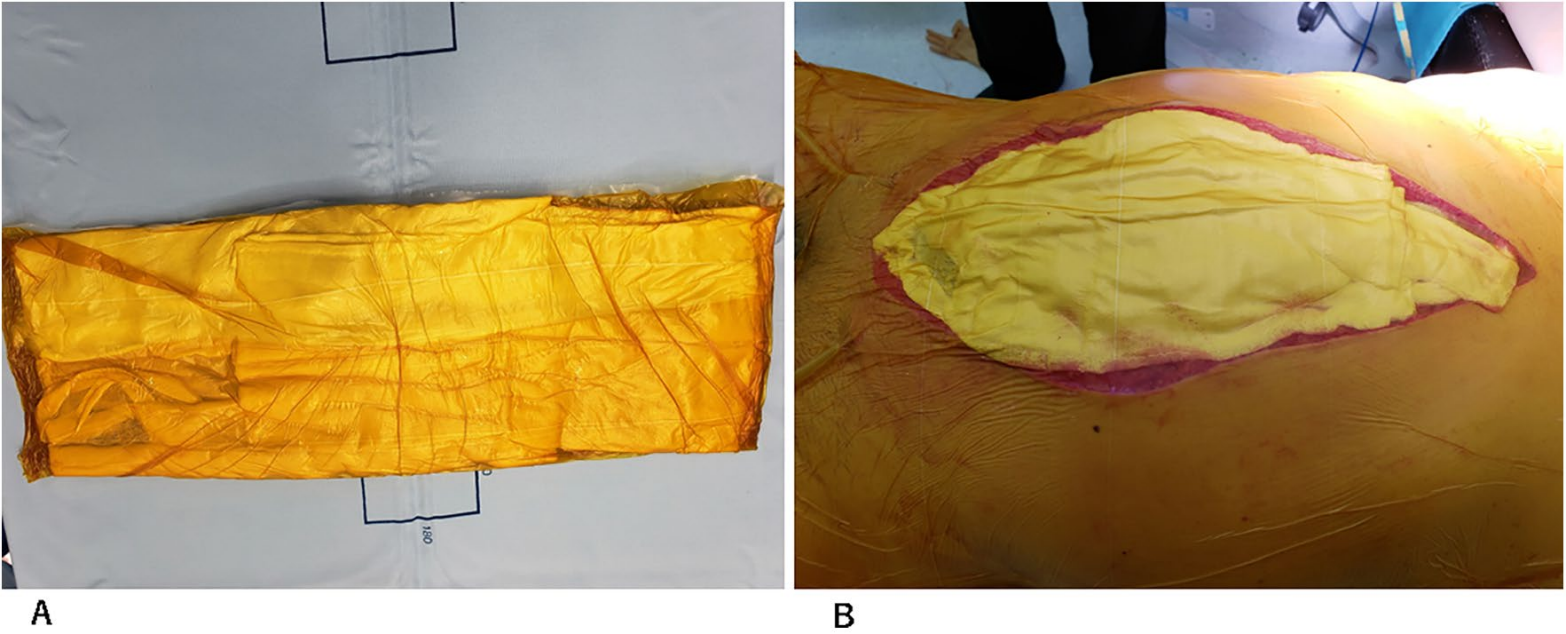
Self-made negative pressure therapy (NPT) system. **A** A large gauze sponge is covered with an incision drape. **B** The large gauze sponge covered with an incision drape is placed on the abdominal wall. In addition, two drains are inserted into the abdominal cavity and controlled by applying negative pressure

OA was indicated when the abdomen could not be closed during the primary operation in all cases. No patients required delayed OA due to the development of ACS after the initial surgery. Patients who required large volumes of fluid infusions and/or blood transfusions before or during surgery, patients with extensive retroperitoneal hematoma, and patients with marked intestinal edema were selected for OA. However, the decision to perform OA rested with the attending surgeon. A large gauze sponge covered with an incise drape was placed on the abdominal wall with a running polypropylene suture (Fig. 1A, B). In addition, two drains were inserted into the abdominal cavity and controlled by applying negative pressure (25 mmHg). In two patients who underwent OA after August 2019, OA was performed using the NPT system (ABTHERA®) when the test laparotomy was performed two to three days after the first surgery.

### ICU management following OA

Renal replacement therapy (RRT) was actively introduced to improve volume overload in some cases. Regarding respiratory management, tracheostomy was performed when long-term ventilator management was required. Upon consultation with a gastrointestinal surgeon, tube feeding was introduced as soon as possible. The optimal timing of definitive abdominal wall closure was decided based on consultations with the gastrointestinal surgeon, and definitive abdominal wall closure surgery was led by the gastrointestinal surgeon. In the non-OA group, we attempted to detect delayed ACS by measuring the intra-abdominal pressure (IAP). Similarly, we attempted to detect secondary ACS after abdominal closure by measuring the IAP in the OA group.

### Statistical analyses

All statistical analyses were performed using the statistical software EZR (Easy R) on R Commander [19]. Continuous variables were expressed as the mean ± standard deviation and compared using the Mann–Whitney *U* test, while categorical variables were expressed as counts and percentages and compared using Fisher’s exact test. For all analyses, statistical significance was set at *p* < 0.05.

## Results

### Preoperative characteristics

The patient’s preoperative characteristics are summarized in Table 1. There were no marked differences in age and sex between the two groups. The OA group included significantly more cases of a preoperative shock than the non-OA group (75.0% vs. 33.3%, *p* = 0.01). The door-to-procedure time was not significantly lower in the OA group than in the non-OA group (*p* = 0.48). The number of patients with Fitzgerald classification [20] III and IV was not significantly different between the two groups, but the OA group had slightly more cases with Fitzgerald classification III and IV than the non-OA group.

**Table 1:** Preoperative characteristics

	Group OA (*n* = 12)	Group non-OA (*n* = 21)	*p* value
Age	75.2 ± 7.7	73.3 ± 11.7	0.82
Female sex	2 (16.6)	8 (38.0)	0.25
Preoperative shock	9 (75.0)	6 (28.5)	0.01
Door to procedure time (h)	3.4 ± 2.7	13.7 ± 33.5	0.48
Fitzgerald classification			
I	1 (8.3)	8 (38.0)	0.10
II	2 (16.6)	7 (33.3)	0.42
III	4 (33.3)	3 (14.2)	0.37
IV	4 (33.3)	2 (9.5)	0.15
Unknown	1(8.3)	1 (4.7)	1.0

### Intraoperative and postoperative outcomes

The intraoperative and postoperative outcomes of the patients are summarized in Table 2. The operation time was significantly longer in the OA group than in the non-OA group (*p* = 0.01). The rate of intraoperative fluid use, bleeding amount, and the need for blood transfusion were significantly higher in the OA group than in the non-OA group (*p* < 0.001).

**Table 2:** Intraoperative and postoperative outcomes

	Group OA (*n* = 12)	Group non-OA (*n* = 21)	*p* value
Procedure			
Open repair	12 (100)	21 (100)	1.0
Operative factor			
Operation time (min)	317.3 ± 76.9	248.0 ± 61.8	0.01
Fluid infusion (min)	5583.6 ± 2069.1	2755.0 ± 841.1	< 0.001
Urine volume (mL)	210.0 ± 265.0	438.7 ± 415.5	0.05
Bleeding (mL)	5553.8 ± 1904.4	1747.7 ± 956.1	< 0.001
Transfusion (mL)	4261.8 ± 1255.3	1810.4 ± 1069.6	< 0.001
Postoperative factor			
Hospital death	6 (50)	3 (14.2)	0.04
RRT	7 (58.3)	3 (14.2)	0.01
Tracheostomy	5 (41.6)	0	0.003
Number of laparotomy			
After 1st operation	1.9 ± 1.8		
Duration of OA (days)	10.3 ± 6.7		
LHS of survivors (days)	69.8 ± 35.7	23.8 ± 22.9	0.003
Home discharge of survivors	3/6 (50)	15/18 (83.3)	0.13

*EVAR* Endovascular aortic repair, *RRT* Renal replacement therapy, *POD* Postoperative day, *LHS* Length of hospital stay

The mortality rate was significantly higher in the OA group than in the non-OA group (50% vs. 14.2%, *p* = 0.04). RRT and tracheostomy rates were also significantly higher in the OA group than in the non-OA group. The duration of OA was 10.3 ± 6.7 days. The length of hospital stay in those who survived was significantly longer in the OA than in the non-OA group (69.8 ± 35.7 days vs. 23.8 ± 22.9 days, *p* = 0.003).

### Postoperative outcomes of the OA group

Postoperative outcomes in the OA group are summarized in Table 3. In fatal cases, the abdomen could not be closed. The number of laparotomies in surviving patients was 2.5 ± 1.1. Definitive abdominal closure was performed using the CS technique in five patients. In two patients, the abdomen was closed more than two weeks after the first operation. Of the six surviving patients in the OA group, tracheostomy was performed in four patients, and tube feeding was also performed in four patients. No patients had an incisional hernia, entero-atmospheric fistula, or graft infection. Five patients were able to be fed orally at the time of discharge. Furthermore, all surviving patients required long-term hospitalization.

**Table 3:** Postoperative outcomes of group OA

Case	Number of laparotomy	Abdominal closure	Outcomes
87y M	0	( −)	POD 0 death
76y M	1	( −)	POD 2 death
83y M	6	( −)	POD 41 death
69y M	0	( −)	POD 0 death
79y F	1	( −)	POD 4 death
89y M	0	( −)	POD 2 death
68y M	4	POD 21, components separation technique	POD 123 TAH
69y F	2	POD 11, components separation technique	POD 104 discharge
65y M	2	POD 5, components separation technique	POD 47 TAH
77y M	2	POD 7, simple closure	POD 43 discharge
75y M	1	POD 3, components separation technique	POD 22 discharge
66y M	4	POD 15 components separation technique	POD 79 TAH

*POD* postoperative day, *TAH* transfer to another hospital

### Case presentation

**Fig. 2:**
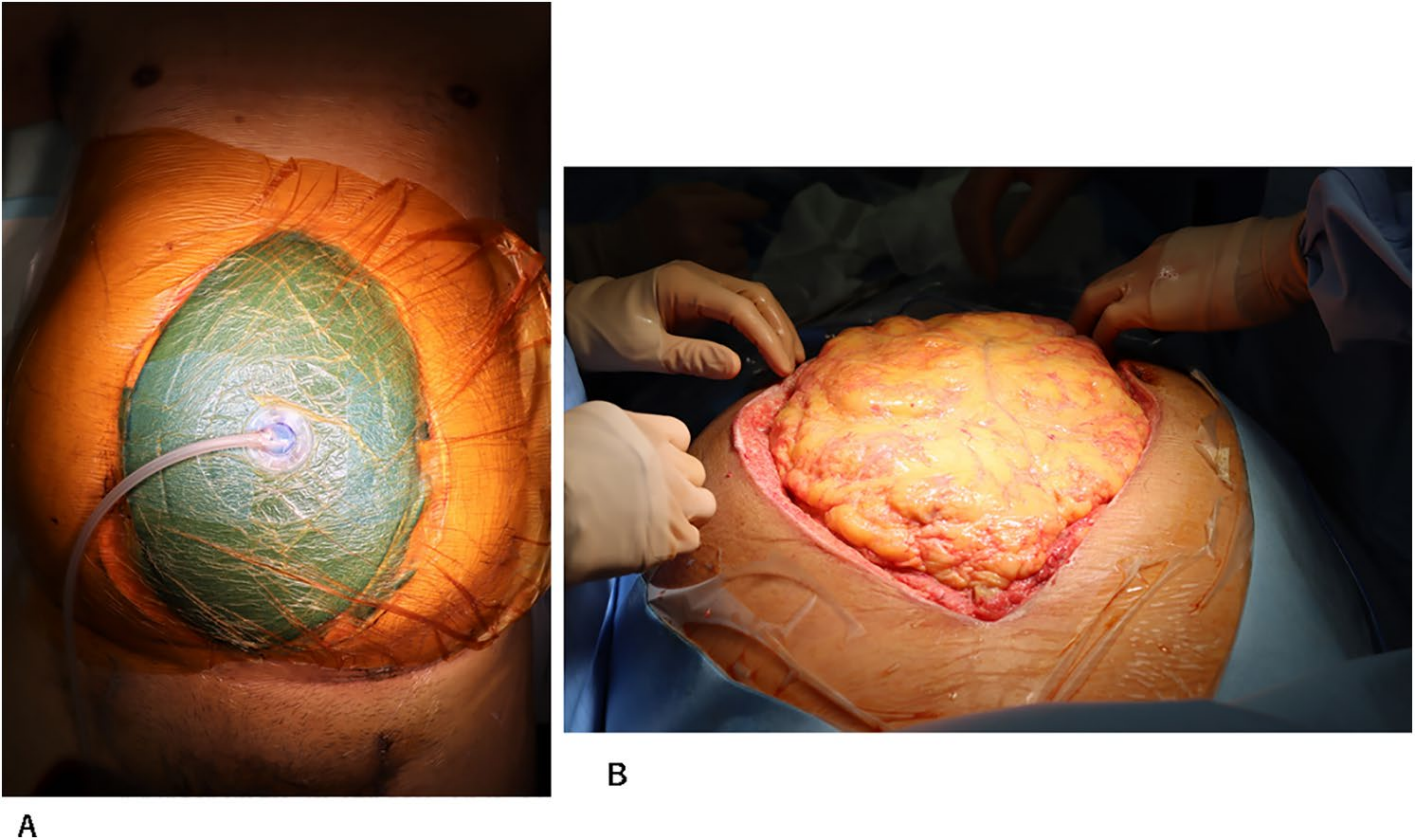
**A** The abdominal condition using the NPT system (ABTHERA®). **B** The abdominal condition with the NPT system (ABTHERA®) removed

**Fig. 3:**
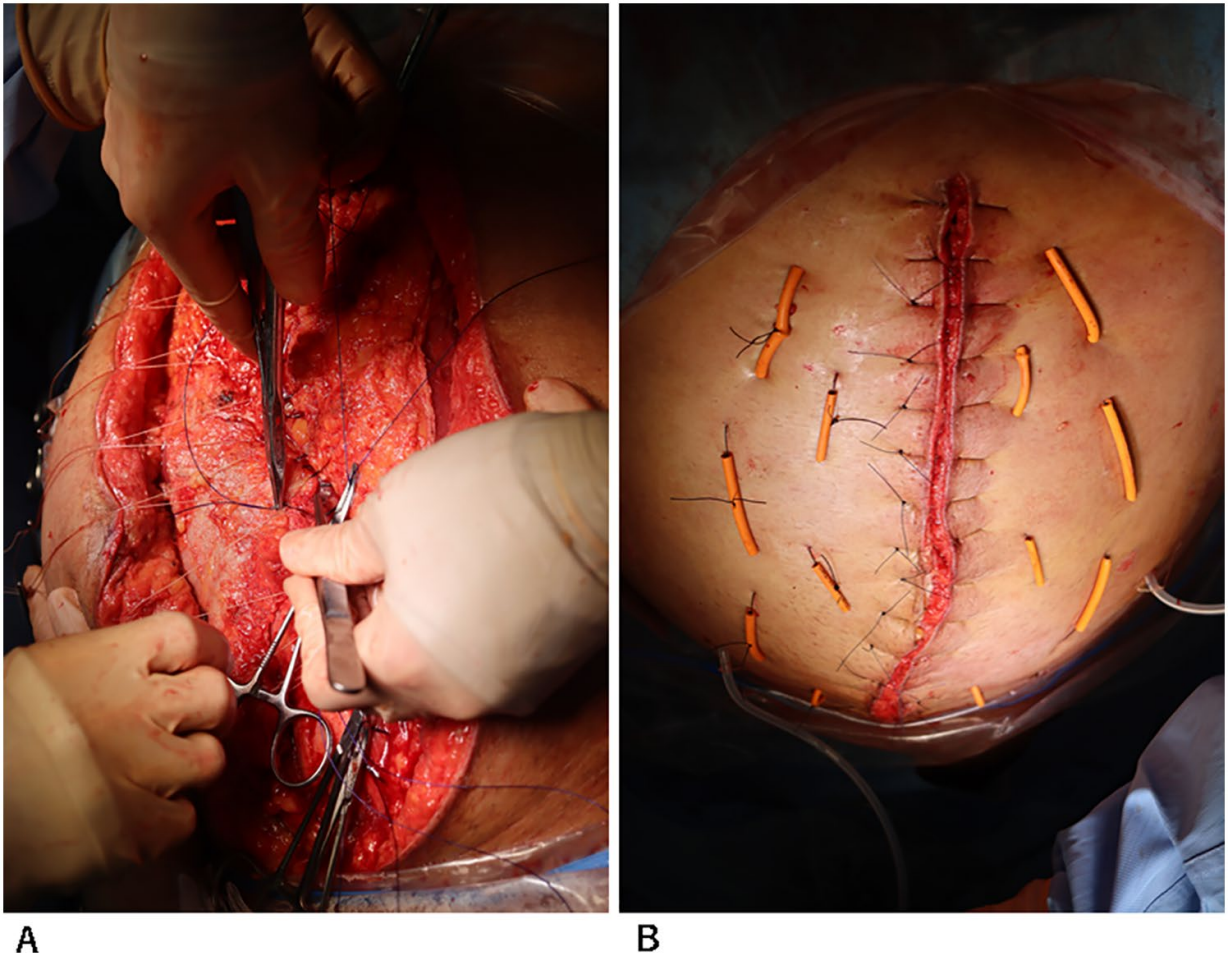
A The abdominal wall was closed using the components separation technique. **B** A retention suture is used

**Fig. 4:**
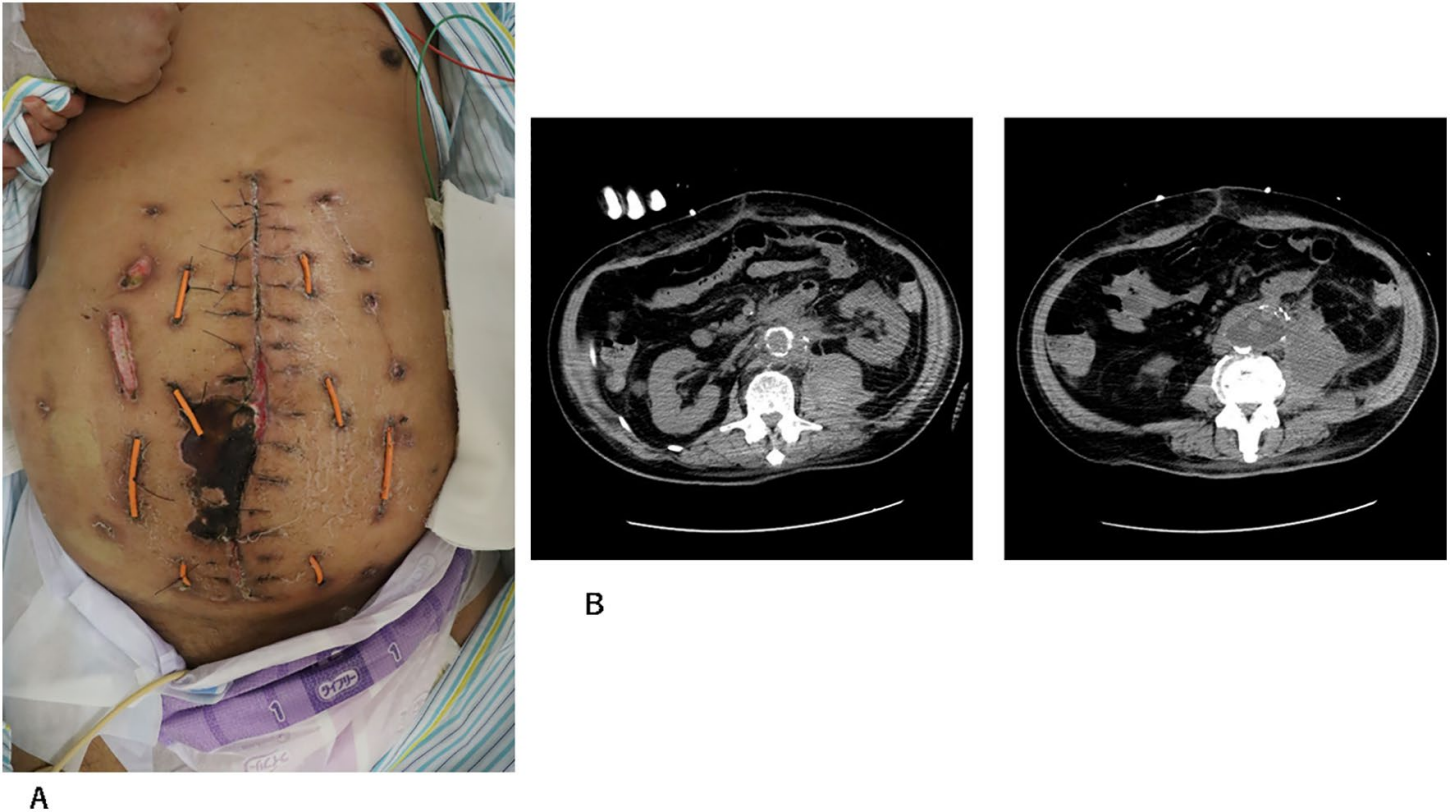
A The abdominal condition 46 days after the first operation. **B** CT findings showed that the fascia was closed, but a retroperitoneum hematoma remained

A 66-year-old man presented with abdominal and back pain. He was diagnosed with Fitzgerald type III rAAA by computed tomography (CT). He was in shock, intratracheally intubated, and immediately transported to the operating room. The aneurysm was resected and reconstructed using a prosthetic graft. Abdominal wall closure was not possible, and OA was performed because of the presence of a massive hematoma in the retroperitoneum and intestinal edema. Test laparotomy was performed the day after the operation, and hemostasis and intestinal conditions were confirmed. NPT (ABTHERA^®^; Kinetic Concepts, Inc., USA) was initiated on day 2 after the first surgery. On day 7 after the first surgery, a third laparotomy and tracheostomy were performed. Definitive abdominal closure was performed 15 days after the initial surgery. Fig. 2A shows the use of the NPT system (ABTHERA®) on the same day. Fig. 2B shows the condition of the abdomen after the removal of the NPT system (ABTHERA®) The abdominal wall was closed using the CS technique (Fig. 3A). A retention suture was then used (Fig. 3B). Figure 4A shows the patient’s abdominal condition 46 days after the first surgery. He had necrosis at the wound edge, but no incisional hernia was observed. Based on the CT findings of the same day, the fascia was closed. However, the retroperitoneum hematoma persisted (Fig. 4B). The patient was transferred to a rehabilitation hospital 79 days after the first surgery.

## Discussion

The incidence of ACS after rAAA surgery is reported to be 3.7–29.0%, and this also increases mortality [12, 13, 21–24]. Prior to 2016, we did not perform OA, instead performing abdominal closure using a retention suture at the initial surgery. Therefore, there was a case of death after rAAA surgery in which we suspected multiple organ failure (MOF) due to ACS as the cause of death. Since 2016, we have actively introduced OA, performing it on 12 patients, and saving 6 patients.

ACS after rAAA surgery is associated with preoperative hypotension, preoperative unconsciousness, perioperative bleeding > 5 L, the need for perioperative blood transfusion, and the need for balloon occlusion [23, 25]. In the present study, the choice to perform OA was left to the discretion of the attending surgeon. However, rates of intraoperative fluid infusion, intraoperative bleeding, and intraoperative blood transfusion were significantly higher in the OA group than in the non-OA group. Acosta et al. reported [26] that patients had better outcomes when OA was initiated during the primary operation than during the second operation. In the present study, OA was initiated during the primary operation, and none of the patients underwent a second operation. Therefore, in the present study, it was considered to be appropriate to judge the condition of the intestinal tract and abdominal cavity while considering the risk factors for ACS.

Multidisciplinary therapy is required for the ICU management of patients with OA. Chabot et al. [27] reported that the primary goal of managing patients with OA is to balance resuscitative efforts with attempts to minimize volume overload and visceral edema, which is essential for optimizing the surgical success of primary fascial closure. Therefore, fluid status management should be performed using RRT if necessary. In the present study, 7 patients (58.3%) in the OA group underwent RRT. In addition, early enteral feeding should be considered [27]. In the present study, tube feeding was performed in four of the six surviving patients in the OA group. Patients with OA may have long-term mechanical ventilation needs and exhibit risk factors for acute respiratory distress syndrome (ARDS) [27]. Tracheostomy was performed in four of the six surviving patients in the OA group.

Multiple temporary abdominal closure (TAC) techniques have been described [3, 13, 28, 29]. These include skin-only closure, retention suture, Bogota bag, mesh/sheet, zipper, artificial burr, and NPT systems. Patients with AAA are elderly and often have comorbidities. Therefore, OA treatment may be longer in these patients than in trauma patients [12, 13]. Furthermore, in rAAA patients, residual hematoma in the retroperitoneum can also be a factor that prolongs OA management and makes abdominal wall closure difficult. The NPT system is useful for long-term OA management. We also used the NPT system for OA management as the TAC method in all the cases. As presented in the case report above, ABTHERA® has been recently introduced as the NPT system. ABTHERA® has a number of advantages over other systems, including preventing viscera adherence to the overlying peritoneum, protecting the bowel and allowing fluid drainage, removing cytokines, promoting wound healing, reducing fascial retention, and preventing abdominal wall involution due to the pulling of wound edges to the center [13, 27, 29, 30].

If the time gap from the first surgery to the definitive abdominal closure is long, the abdominal closure rate may decrease [10, 12]. Miller et al. reported [10] that delayed primary fascial closure within eight days of the first operation was associated with the best outcomes. However, as mentioned above, in some cases, OA is performed long after the initial rAAA surgery, necessitating specific procedures for definitive abdominal closure. The CS technique has been used for the repair of complex midline ventral hernia [15, 16]. Rasilainen et al. reported [17] that the CS technique contributed to successful primary fascial closure in patients who received OA. In this technique, by widening the space between the internal abdominal oblique muscle and the abdominal lateral muscle, the passive distance of the rectus abdominis muscle can be increased and the abdomen can be closed. There are few reports of cases in which the CS technique was used to close the abdominal wall of patients who received OA after rAAA surgery. In the present study, the CS technique was used in five of the six surviving patients in the OA group, and abdominal wall closure was successful. The CS technique may thus be useful for achieving definitive abdominal wall closure in patients who undergo long-term OA, such as after rAAA surgery.

### Limitation

This study was limited by being carried out at a single institution in a small cohort. A large cohort study is thus needed to clarify the usefulness of the NPT system and CS technique.

## Conclusions

The NPT system and CS technique may be useful for achieving definitive abdominal wall closure in patients who undergo long-term OA after rAAA surgery.
